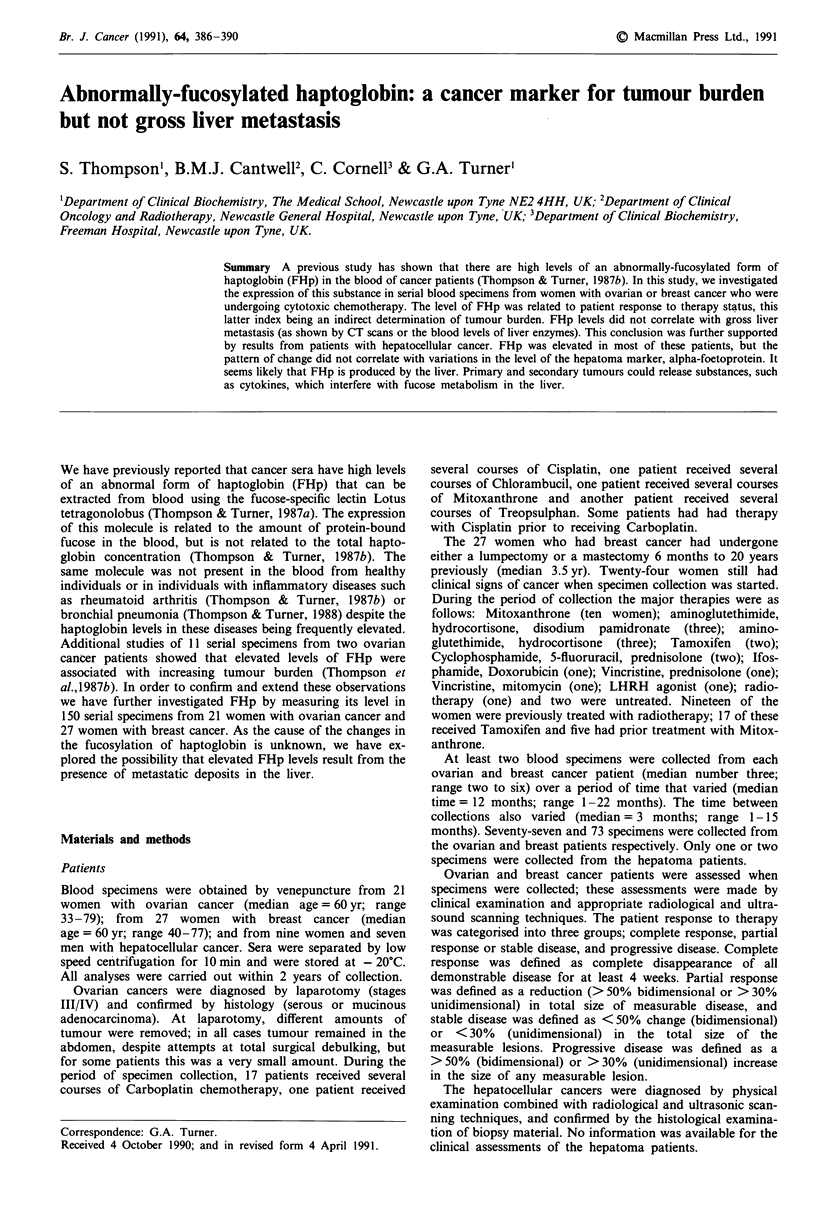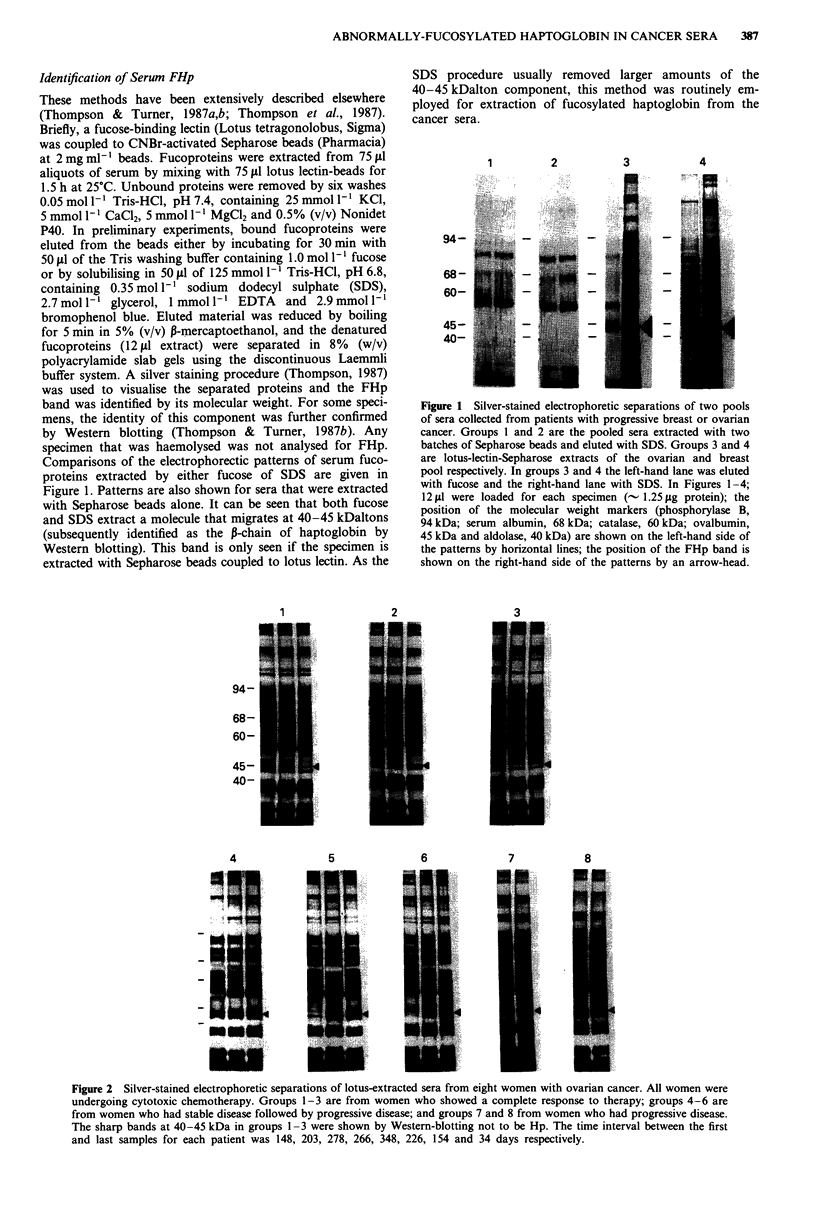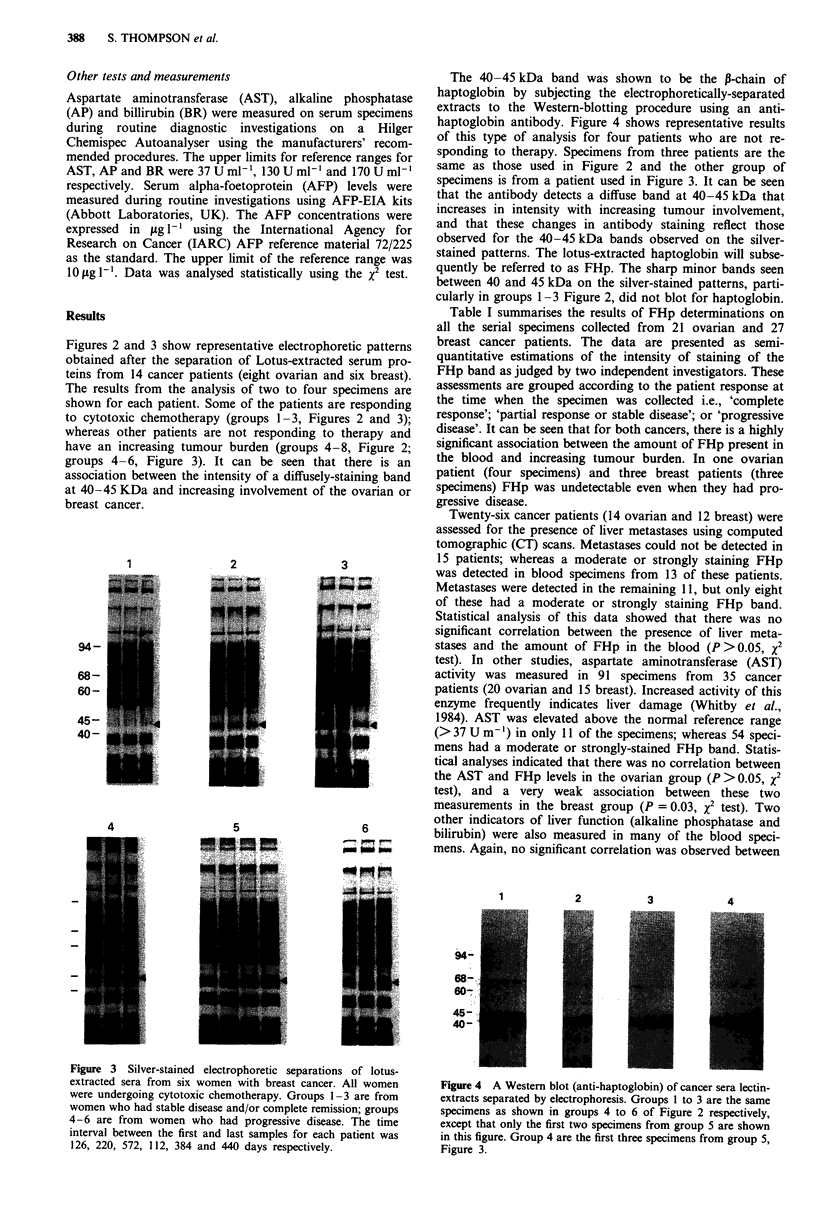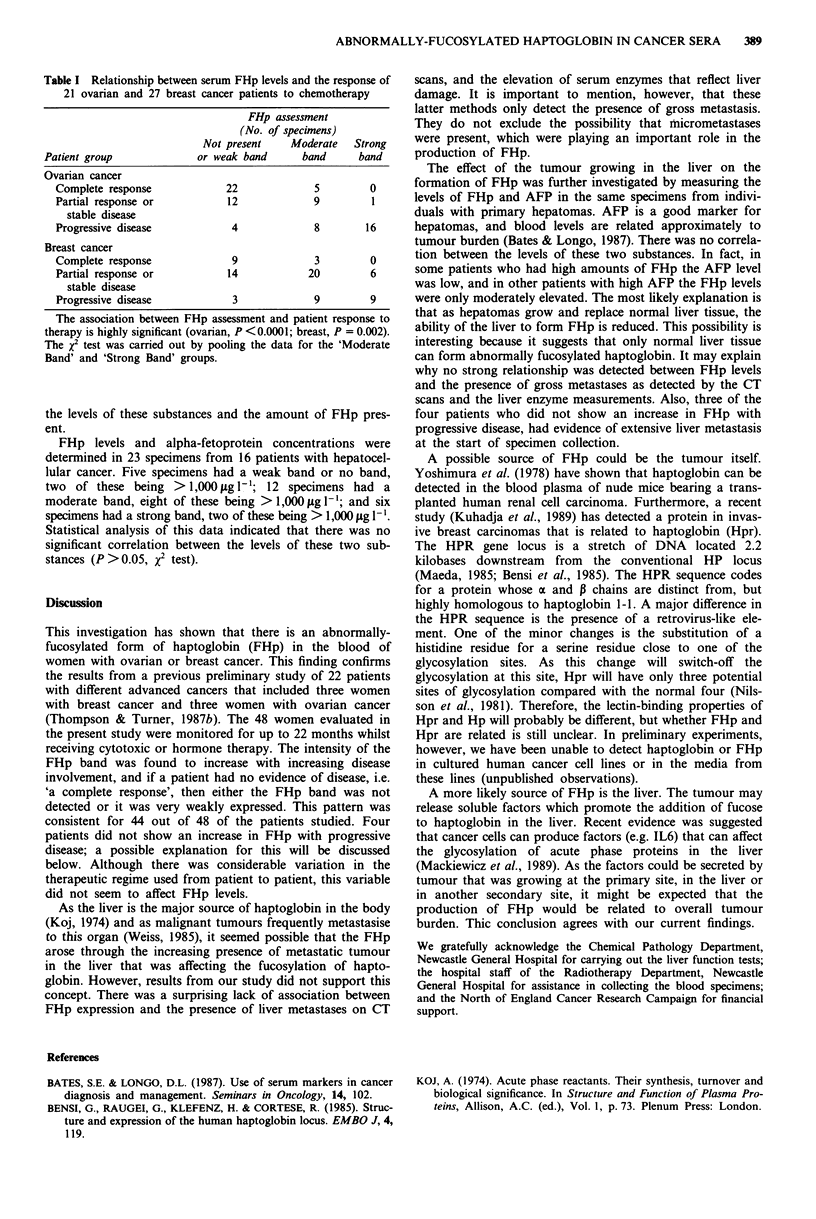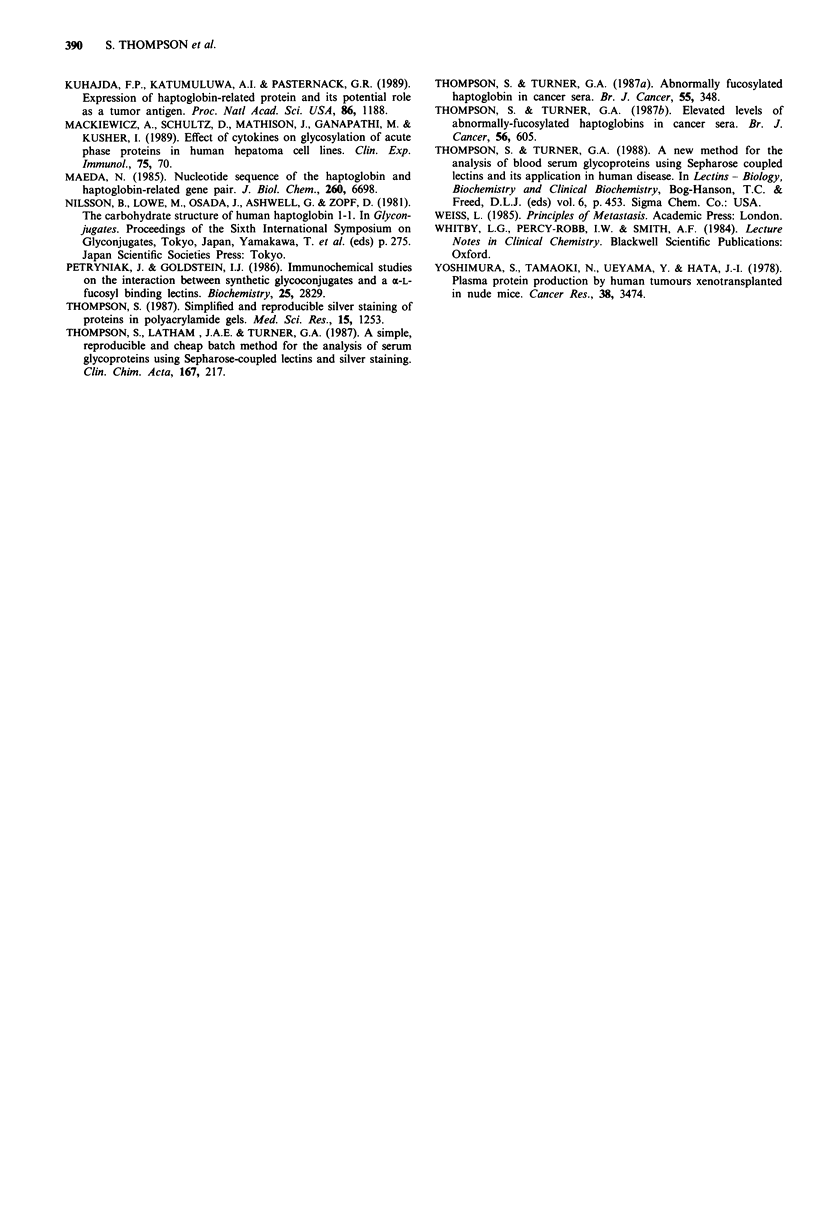# Abnormally-fucosylated haptoglobin: a cancer marker for tumour burden but not gross liver metastasis.

**DOI:** 10.1038/bjc.1991.314

**Published:** 1991-08

**Authors:** S. Thompson, B. M. Cantwell, C. Cornell, G. A. Turner

**Affiliations:** Department of Clinical Biochemistry, Medical School, Newcastle upon Tyne, UK.

## Abstract

**Images:**


Br. J. Cancer (1991), 64, 386-390 © Macmillan Press Ltd., 1991

### Abnormally-fucosylated haptoglobin: a cancer marker for tumour burden but not gross liver metastasis

S. Thompson[1], B.M.J. Cantwell[2], C. Cornell[3] & G.A. Turner[1]

[1]*Department of Clinical Biochemistry, The Medical School, Newcastle upon Tyne NE2 4HH, UK;* [2]*Department of Clinical Oncology and Radiotherapy, Newcastle General Hospital, Newcastle upon Tyne, UK;* [3]*Department of Clinical Biochemistry, Freeman Hospital, Newcastle upon Tyne, UK.*

**Summary** A previous study has shown that there are high levels of an abnormally-fucosylated form of haptoglobin (FHp) in the blood of cancer patients (Thompson & Turner, 1987*b*). In this study, we investigated the expression of this substance in serial blood specimens from women with ovarian or breast cancer who were undergoing cytotoxic chemotherapy. The level of FHp was related to patient response to therapy status, this latter index being an indirect determination of tumour burden. FHp levels did not correlate with gross liver metastasis (as shown by CT scans or the blood levels of liver enzymes). This conclusion was further supported by results from patients with hepatocellular cancer. FHp was elevated in most of these patients, but the pattern of change did not correlate with variations in the level of the hepatoma marker, alpha-foetoprotein. It seems likely that FHp is produced by the liver. Primary and secondary tumours could release substances, such as cytokines, which interfere with fucose metabolism in the liver.

We have previously reported that cancer sera have high levels of an abnormal form of haptoglobin (FHp) that can be extracted from blood using the fucose-specific lectin Lotus tetragonolobus (Thompson & Turner, 1987*a*). The expression of this molecule is related to the amount of protein-bound fucose in the blood, but is not related to the total haptoglobin concentration (Thompson & Turner, 1987*b*). The same molecule was not present in the blood from healthy individuals or in individuals with inflammatory diseases such as rheumatoid arthritis (Thompson & Turner, 1987*b*) or bronchial pneumonia (Thompson & Turner, 1988) despite the haptoglobin levels in these diseases being frequently elevated. Additional studies of 11 serial specimens from two ovarian cancer patients showed that elevated levels of FHp were associated with increasing tumour burden (Thompson *et al.*,1987*b*). In order to confirm and extend these observations we have further investigated FHp by measuring its level in 150 serial specimens from 21 women with ovarian cancer and 27 women with breast cancer. As the cause of the changes in the fucosylation of haptoglobin is unknown, we have explored the possibility that elevated FHp levels result from the presence of metastatic deposits in the liver.

### Materials and methods

### *Patients*

Blood specimens were obtained by venepuncture from 21 women with ovarian cancer (median age = 60 yr; range 33-79); from 27 women with breast cancer (median age = 60 yr; range 40-77); and from nine women and seven men with hepatocellular cancer. Sera were separated by low speed centrifugation for 10 min and were stored at − 20°C. All analyses were carried out within 2 years of collection.

Ovarian cancers were diagnosed by laparotomy (stages III/IV) and confirmed by histology (serous or mucinous adenocarcinoma). At laparotomy, different amounts of tumour were removed; in all cases tumour remained in the abdomen, despite attempts at total surgical debulking, but for some patients this was a very small amount. During the period of specimen collection, 17 patients received several courses of Carboplatin chemotherapy, one patient received several courses of Cisplatin, one patient received several courses of Chlorambucil, one patient received several courses of Mitoxanthrone and another patient received several courses of Treopsulphan. Some patients had had therapy with Cisplatin prior to receiving Carboplatin.

The 27 women who had breast cancer had undergone either a lumpectomy or a mastectomy 6 months to 20 years previously (median 3.5 yr). Twenty-four women still had clinical signs of cancer when specimen collection was started. During the period of collection the major therapies were as follows: Mitoxanthrone (ten women); aminoglutethimide, hydrocortisone, disodium pamidronate (three); aminoglutethimide, hydrocortisone (three); Tamoxifen (two); Cyclophosphamide, 5-fluoruracil, prednisolone (two); Ifosphamide, Doxorubicin (one); Vincristine, prednisolone (one); Vincristine, mitomycin (one); LHRH agonist (one); radiotherapy (one) and two were untreated. Nineteen of the women were previously treated with radiotherapy; 17 of these received Tamoxifen and five had prior treatment with Mitoxanthrone.

At least two blood specimens were collected from each ovarian and breast cancer patient (median number three; range two to six) over a period of time that varied (median time = 12 months; range 1-22 months). The time between collections also varied (median = 3 months; range 1-15 months). Seventy-seven and 73 specimens were collected from the ovarian and breast patients respectively. Only one or two specimens were collected from the hepatoma patients.

Ovarian and breast cancer patients were assessed when specimens were collected; these assessments were made by clinical examination and appropriate radiological and ultrasound scanning techniques. The patient response to therapy was categorised into three groups; complete response, partial response or stable disease, and progressive disease. Complete response was defined as complete disappearance of all demonstrable disease for at least 4 weeks. Partial response was defined as a reduction (> 50% bidimensional or > 30% unidimensional) in total size of measurable disease, and stable disease was defined as < 50% change (bidimensional) or < 30% (unidimensional) in the total size of the measurable lesions. Progressive disease was defined as a > 50% (bidimensional) or > 30% (unidimensional) increase in the size of any measurable lesion.

The hepatocellular cancers were diagnosed by physical examination combined with radiological and ultrasonic scanning techniques, and confirmed by the histological examination of biopsy material. No information was available for the clinical assessments of the hepatoma patients.

Correspondence: G.A. Turner.
Received 4 October 1990; and in revised form 4 April 1991.

*Identification of Serum FHp*

These methods have been extensively described elsewhere (Thompson & Turner, 1987*a,b*; Thompson *et al.*, 1987). Briefly, a fucose-binding lectin (Lotus tetragonolobus, Sigma) was coupled to CNBr-activated Sepharose beads (Pharmacia) at 2 mg $ml^{-1}$ beads. Fucoproteins were extracted from 75 µl aliquots of serum by mixing with 75 µl lotus lectin-beads for 1.5 h at 25°C. Unbound proteins were removed by six washes 0.05 mol $l^{-1}$ Tris-HCl, pH 7.4, containing 25 mmol $l^{-1}$ KCl, 5 mmol $l^{-1}$ $CaCl_2$, 5 mmol $l^{-1}$ $MgCl_2$ and 0.5% (v/v) Nonidet P40. In preliminary experiments, bound fucoproteins were eluted from the beads either by incubating for 30 min with 50 µl of the Tris washing buffer containing 1.0 mol $l^{-1}$ fucose or by solubilising in 50 µl of 125 mmol $l^{-1}$ Tris-HCl, pH 6.8, containing 0.35 mol $l^{-1}$ sodium dodecyl sulphate (SDS), 2.7 mol $l^{-1}$ glycerol, 1 mmol $l^{-1}$ EDTA and 2.9 mmol $l^{-1}$ bromophenol blue. Eluted material was reduced by boiling for 5 min in 5% (v/v) β-mercaptoethanol, and the denatured fucoproteins (12 µl extract) were separated in 8% (w/v) polyacrylamide slab gels using the discontinuous Laemmli buffer system. A silver staining procedure (Thompson, 1987) was used to visualise the separated proteins and the FHp band was identified by its molecular weight. For some specimens, the identity of this component was further confirmed by Western blotting (Thompson & Turner, 1987*b*). Any specimen that was haemolysed was not analysed for FHp. Comparisons of the electrophorectic patterns of serum fucoproteins extracted by either fucose of SDS are given in Figure 1. Patterns are also shown for sera that were extracted with Sepharose beads alone. It can be seen that both fucose and SDS extract a molecule that migrates at 40–45 kDaltons (subsequently identified as the β-chain of haptoglobin by Western blotting). This band is only seen if the specimen is extracted with Sepharose beads coupled to lotus lectin. As the SDS procedure usually removed larger amounts of the 40–45 kDalton component, this method was routinely employed for extraction of fucosylated haptoglobin from the cancer sera.

**Figure 1** Silver-stained electrophoretic separations of two pools of sera collected from patients with progressive breast or ovarian cancer. Groups 1 and 2 are the pooled sera extracted with two batches of Sepharose beads and eluted with SDS. Groups 3 and 4 are lotus-lectin-Sepharose extracts of the ovarian and breast pool respectively. In groups 3 and 4 the left-hand lane was eluted with fucose and the right-hand lane with SDS. In Figures 1–4; 12 µl were loaded for each specimen (~ 1.25 µg protein); the position of the molecular weight markers (phosphorylase B, 94 kDa; serum albumin, 68 kDa; catalase, 60 kDa; ovalbumin, 45 kDa and aldolase, 40 kDa) are shown on the left-hand side of the patterns by horizontal lines; the position of the FHp band is shown on the right-hand side of the patterns by an arrow-head.

**Figure 2** Silver-stained electrophoretic separations of lotus-extracted sera from eight women with ovarian cancer. All women were undergoing cytotoxic chemotherapy. Groups 1–3 are from women who showed a complete response to therapy; groups 4–6 are from women who had stable disease followed by progressive disease; and groups 7 and 8 from women who had progressive disease. The sharp bands at 40–45 kDa in groups 1–3 were shown by Western-blotting not to be Hp. The time interval between the first and last samples for each patient was 148, 203, 278, 266, 348, 226, 154 and 34 days respectively.

*Other tests and measurements*

Aspartate aminotransferase (AST), alkaline phosphatase (AP) and billirubin (BR) were measured on serum specimens during routine diagnostic investigations on a Hilger Chemispec Autoanalyser using the manufacturers' recommended procedures. The upper limits for reference ranges for AST, AP and BR were 37 U $ml^{-1}$, 130 U $ml^{-1}$ and 170 U $ml^{-1}$ respectively. Serum alpha-foetoprotein (AFP) levels were measured during routine investigations using AFP-EIA kits (Abbott Laboratories, UK). The AFP concentrations were expressed in $\mu g\,l^{-1}$ using the International Agency for Research on Cancer (IARC) AFP reference material 72/225 as the standard. The upper limit of the reference range was 10 $\mu g\,l^{-1}$. Data was analysed statistically using the $\chi^2$ test.

### Results

Figures 2 and 3 show representative electrophoretic patterns obtained after the separation of Lotus-extracted serum proteins from 14 cancer patients (eight ovarian and six breast). The results from the analysis of two to four specimens are shown for each patient. Some of the patients are responding to cytotoxic chemotherapy (groups 1–3, Figures 2 and 3); whereas other patients are not responding to therapy and have an increasing tumour burden (groups 4–8, Figure 2; groups 4–6, Figure 3). It can be seen that there is an association between the intensity of a diffusely-staining band at 40–45 KDa and increasing involvement of the ovarian or breast cancer.

**Figure 3** Silver-stained electrophoretic separations of lotus-extracted sera from six women with breast cancer. All women were undergoing cytotoxic chemotherapy. Groups 1–3 are from women who had stable disease and/or complete remission; groups 4–6 are from women who had progressive disease. The time interval between the first and last samples for each patient was 126, 220, 572, 112, 384 and 440 days respectively.

The 40–45 kDa band was shown to be the $\beta$-chain of haptoglobin by subjecting the electrophoretically-separated extracts to the Western-blotting procedure using an anti-haptoglobin antibody. Figure 4 shows representative results of this type of analysis for four patients who are not responding to therapy. Specimens from three patients are the same as those used in Figure 2 and the other group of specimens is from a patient used in Figure 3. It can be seen that the antibody detects a diffuse band at 40–45 kDa that increases in intensity with increasing tumour involvement, and that these changes in antibody staining reflect those observed for the 40–45 kDa bands observed on the silver-stained patterns. The lotus-extracted haptoglobin will subsequently be referred to as FHp. The sharp minor bands seen between 40 and 45 kDa on the silver-stained patterns, particularly in groups 1–3 Figure 2, did not blot for haptoglobin.

Table I summarises the results of FHp determinations on all the serial specimens collected from 21 ovarian and 27 breast cancer patients. The data are presented as semi-quantitative estimations of the intensity of staining of the FHp band as judged by two independent investigators. These assessments are grouped according to the patient response at the time when the specimen was collected i.e., 'complete response'; 'partial response or stable disease'; or 'progressive disease'. It can be seen that for both cancers, there is a highly significant association between the amount of FHp present in the blood and increasing tumour burden. In one ovarian patient (four specimens) and three breast patients (three specimens) FHp was undetectable even when they had progressive disease.

Twenty-six cancer patients (14 ovarian and 12 breast) were assessed for the presence of liver metastases using computed tomographic (CT) scans. Metastases could not be detected in 15 patients; whereas a moderate or strongly staining FHp was detected in blood specimens from 13 of these patients. Metastases were detected in the remaining 11, but only eight of these had a moderate or strongly staining FHp band. Statistical analysis of this data showed that there was no significant correlation between the presence of liver metastases and the amount of FHp in the blood ($P>0.05$, $\chi^2$ test). In other studies, aspartate aminotransferase (AST) activity was measured in 91 specimens from 35 cancer patients (20 ovarian and 15 breast). Increased activity of this enzyme frequently indicates liver damage (Whitby *et al.*, 1984). AST was elevated above the normal reference range ($>37$ U $m^{-1}$) in only 11 of the specimens; whereas 54 specimens had a moderate or strongly-stained FHp band. Statistical analyses indicated that there was no correlation between the AST and FHp levels in the ovarian group ($P>0.05$, $\chi^2$ test), and a very weak association between these two measurements in the breast group ($P=0.03$, $\chi^2$ test). Two other indicators of liver function (alkaline phosphatase and bilirubin) were also measured in many of the blood specimens. Again, no significant correlation was observed between

**Figure 4** A Western blot (anti-haptoglobin) of cancer sera lectin-extracts separated by electrophoresis. Groups 1 to 3 are the same specimens as shown in groups 4 to 6 of Figure 2 respectively, except that only the first two specimens from group 5 are shown in this figure. Group 4 are the first three specimens from group 5, Figure 3.

**Table I** Relationship between serum FHp levels and the response of 21 ovarian and 27 breast cancer patients to chemotherapy

| Patient group | *FHp assessment (No. of specimens)* Not present or weak band | Moderate band | Strong band |
|---|---|---|---|
| Ovarian cancer | | | |
| Complete response | 22 | 5 | 0 |
| Partial response or stable disease | 12 | 9 | 1 |
| Progressive disease | 4 | 8 | 16 |
| Breast cancer | | | |
| Complete response | 9 | 3 | 0 |
| Partial response or stable disease | 14 | 20 | 6 |
| Progressive disease | 3 | 9 | 9 |

The association between FHp assessment and patient response to therapy is highly significant (ovarian, $P < 0.0001$; breast, $P = 0.002$). The $\chi^2$ test was carried out by pooling the data for the 'Moderate Band' and 'Strong Band' groups.

the levels of these substances and the amount of FHp present.

FHp levels and alpha-fetoprotein concentrations were determined in 23 specimens from 16 patients with hepatocellular cancer. Five specimens had a weak band or no band, two of these being $> 1{,}000\ \mu g\ l^{-1}$; 12 specimens had a moderate band, eight of these being $> 1{,}000\ \mu g\ l^{-1}$; and six specimens had a strong band, two of these being $> 1{,}000\ \mu g\ l^{-1}$. Statistical analysis of this data indicated that there was no significant correlation between the levels of these two substances ($P > 0.05$, $\chi^2$ test).

### Discussion

This investigation has shown that there is an abnormally-fucosylated form of haptoglobin (FHp) in the blood of women with ovarian or breast cancer. This finding confirms the results from a previous preliminary study of 22 patients with different advanced cancers that included three women with breast cancer and three women with ovarian cancer (Thompson & Turner, 1987*b*). The 48 women evaluated in the present study were monitored for up to 22 months whilst receiving cytotoxic or hormone therapy. The intensity of the FHp band was found to increase with increasing disease involvement, and if a patient had no evidence of disease, i.e. 'a complete response', then either the FHp band was not detected or it was very weakly expressed. This pattern was consistent for 44 out of 48 of the patients studied. Four patients did not show an increase in FHp with progressive disease; a possible explanation for this will be discussed below. Although there was considerable variation in the therapeutic regime used from patient to patient, this variable did not seem to affect FHp levels.

As the liver is the major source of haptoglobin in the body (Koj, 1974) and as malignant tumours frequently metastasise to this organ (Weiss, 1985), it seemed possible that the FHp arose through the increasing presence of metastatic tumour in the liver that was affecting the fucosylation of haptoglobin. However, results from our study did not support this concept. There was a surprising lack of association between FHp expression and the presence of liver metastases on CT scans, and the elevation of serum enzymes that reflect liver damage. It is important to mention, however, that these latter methods only detect the presence of gross metastasis. They do not exclude the possibility that micrometastases were present, which were playing an important role in the production of FHp.

The effect of the tumour growing in the liver on the formation of FHp was further investigated by measuring the levels of FHp and AFP in the same specimens from individuals with primary hepatomas. AFP is a good marker for hepatomas, and blood levels are related approximately to tumour burden (Bates & Longo, 1987). There was no correlation between the levels of these two substances. In fact, in some patients who had high amounts of FHp the AFP level was low, and in other patients with high AFP the FHp levels were only moderately elevated. The most likely explanation is that as hepatomas grow and replace normal liver tissue, the ability of the liver to form FHp is reduced. This possibility is interesting because it suggests that only normal liver tissue can form abnormally fucosylated haptoglobin. It may explain why no strong relationship was detected between FHp levels and the presence of gross metastases as detected by the CT scans and the liver enzyme measurements. Also, three of the four patients who did not show an increase in FHp with progressive disease, had evidence of extensive liver metastasis at the start of specimen collection.

A possible source of FHp could be the tumour itself. Yoshimura *et al.* (1978) have shown that haptoglobin can be detected in the blood plasma of nude mice bearing a transplanted human renal cell carcinoma. Furthermore, a recent study (Kuhadja *et al.*, 1989) has detected a protein in invasive breast carcinomas that is related to haptoglobin (Hpr). The HPR gene locus is a stretch of DNA located 2.2 kilobases downstream from the conventional HP locus (Maeda, 1985; Bensi *et al.*, 1985). The HPR sequence codes for a protein whose $\alpha$ and $\beta$ chains are distinct from, but highly homologous to haptoglobin 1-1. A major difference in the HPR sequence is the presence of a retrovirus-like element. One of the minor changes is the substitution of a histidine residue for a serine residue close to one of the glycosylation sites. As this change will switch-off the glycosylation at this site, Hpr will have only three potential sites of glycosylation compared with the normal four (Nilsson *et al.*, 1981). Therefore, the lectin-binding properties of Hpr and Hp will probably be different, but whether FHp and Hpr are related is still unclear. In preliminary experiments, however, we have been unable to detect haptoglobin or FHp in cultured human cancer cell lines or in the media from these lines (unpublished observations).

A more likely source of FHp is the liver. The tumour may release soluble factors which promote the addition of fucose to haptoglobin in the liver. Recent evidence was suggested that cancer cells can produce factors (e.g. IL6) that can affect the glycosylation of acute phase proteins in the liver (Mackiewicz *et al.*, 1989). As the factors could be secreted by tumour that was growing at the primary site, in the liver or in another secondary site, it might be expected that the production of FHp would be related to overall tumour burden. Thic conclusion agrees with our current findings.

We gratefully acknowledge the Chemical Pathology Department, Newcastle General Hospital for carrying out the liver function tests; the hospital staff of the Radiotherapy Department, Newcastle General Hospital for assistance in collecting the blood specimens; and the North of England Cancer Research Campaign for financial support.

### References

BATES, S.E. & LONGO, D.L. (1987). Use of serum markers in cancer diagnosis and management. *Seminars in Oncology*, **14**, 102.

BENSI, G., RAUGEI, G., KLEFENZ, H. & CORTESE, R. (1985). Structure and expression of the human haptoglobin locus. *EMBO J*, **4**, 119.

KOJ, A. (1974). Acute phase reactants. Their synthesis, turnover and biological significance. In *Structure and Function of Plasma Proteins*, Allison, A.C. (ed.), Vol. 1, p. 73. Plenum Press: London.

KUHAJDA, F.P., KATUMULUWA, A.I. & PASTERNACK, G.R. (1989). Expression of haptoglobin-related protein and its potential role as a tumor antigen. *Proc. Natl Acad. Sci. USA*, **86**, 1188.

MACKIEWICZ, A., SCHULTZ, D., MATHISON, J., GANAPATHI, M. & KUSHER, I. (1989). Effect of cytokines on glycosylation of acute phase proteins in human hepatoma cell lines. *Clin. Exp. Immunol.*, **75**, 70.

MAEDA, N. (1985). Nucleotide sequence of the haptoglobin and haptoglobin-related gene pair. *J. Biol. Chem.*, **260**, 6698.

NILSSON, B., LOWE, M., OSADA, J., ASHWELL, G. & ZOPF, D. (1981). The carbohydrate structure of human haptoglobin 1-1. In *Glyconjugates*. Proceedings of the Sixth International Symposium on Glyconjugates, Tokyo, Japan, Yamakawa, T. *et al.* (eds) p. 275. Japan Scientific Societies Press: Tokyo.

PETRYNIAK, J. & GOLDSTEIN, I.J. (1986). Immunochemical studies on the interaction between synthetic glycoconjugates and a α-L-fucosyl binding lectins. *Biochemistry*, **25**, 2829.

THOMPSON, S. (1987). Simplified and reproducible silver staining of proteins in polyacrylamide gels. *Med. Sci. Res.*, **15**, 1253.

THOMPSON, S., LATHAM , J.A.E. & TURNER, G.A. (1987). A simple, reproducible and cheap batch method for the analysis of serum glycoproteins using Sepharose-coupled lectins and silver staining. *Clin. Chim. Acta*, **167**, 217.

THOMPSON, S. & TURNER, G.A. (1987*a*). Abnormally fucosylated haptoglobin in cancer sera. *Br. J. Cancer*, **55**, 348.

THOMPSON, S. & TURNER, G.A. (1987*b*). Elevated levels of abnormally-fucosylated haptoglobins in cancer sera. *Br. J. Cancer*, **56**, 605.

THOMPSON, S. & TURNER, G.A. (1988). A new method for the analysis of blood serum glycoproteins using Sepharose coupled lectins and its application in human disease. In *Lectins – Biology, Biochemistry and Clinical Biochemistry*, Bog-Hanson, T.C. & Freed, D.L.J. (eds) vol. 6, p. 453. Sigma Chem. Co.: USA.

WEISS, L. (1985). *Principles of Metastasis*. Academic Press: London.

WHITBY, L.G., PERCY-ROBB, I.W. & SMITH, A.F. (1984). *Lecture Notes in Clinical Chemistry*. Blackwell Scientific Publications: Oxford.

YOSHIMURA, S., TAMAOKI, N., UEYAMA, Y. & HATA, J.-I. (1978). Plasma protein production by human tumours xenotransplanted in nude mice. *Cancer Res.*, **38**, 3474.